# Burst estimation through atomic decomposition (BEAD): A toolbox to find oscillatory bursts in brain signals

**DOI:** 10.1162/IMAG.a.86

**Published:** 2025-07-21

**Authors:** Abhishek Anand, Chandra Murthy, Supratim Ray

**Affiliations:** Electrical Communication Engineering, Indian Institute of Science, Bengaluru, Karnataka, India; Centre for Neuroscience, Indian Institute of Science, Bengaluru, Karnataka, India

**Keywords:** burst detection, gamma, local field potential, matching pursuit, compressed sensing, overcomplete representations

## Abstract

Recent studies have shown that brain signals often show oscillatory bursts of short durations, which have been linked to various aspects of computation and behavior. Traditional methods often use direct spectral estimators to estimate the power of brain signals in spectral and temporal domains, from which bursts are identified. However, direct spectral estimators are known to be noisy, such that even stable oscillations may appear bursty. We have previously shown that the Matching Pursuit (MP) algorithm, which uses a large overcomplete dictionary of basis functions (called “atoms”) to decompose the signal directly in the time domain, partly addresses this concern and robustly finds long bursts in synthetic as well as real data. However, MP is a greedy algorithm that can give non-optimal solutions and requires a large-sized dictionary. To address these concerns, we extended two other algorithms—orthogonal MP (OMP) and OMP using Multiscale Adaptive Gabor Expansion (OMP-MAGE), to perform burst duration estimation. We also develop a novel algorithm, called OMP using Gabor Expansion with Atom Reassignment (OMP-GEAR). These algorithms overcome the limitations of MP and can work with a significantly smaller dictionary size. We find that, in synthetic data, OMP, OMP-MAGE, and OMP-GEAR converge faster than MP. Also, OMP-MAGE and OMP-GEAR outperform both MP and OMP when the dictionary size is small. Finally, OMP-GEAR significantly outperforms OMP-MAGE when the bursts are overlapping. Importantly, the burst durations obtained using MP and OMP with a very large-sized dictionary are comparable with that obtained using OMP-MAGE with a much smaller-sized dictionary in real data obtained from two monkeys passively viewing static gratings which induced gamma bursts in the primary visual cortex. OMP-GEAR yields slightly smaller burst durations, but all the estimated burst durations are still significantly larger than the duration estimated using traditional methods. These results suggest that gamma bursts are longer than previously reported. Raw data from two monkeys, as well as codes for both traditional and new methods, are publicly available as part of this toolbox.

## 1 Introduction

Oscillations in different frequency bands such as beta (13–26 Hz) and gamma (30–80 Hz) in brain signals are linked to various brain functions (Buzsáki, 2006). For example, both actual and imagery movements are associated with beta desynchronization (Mcfarland et al., 2000). Different regions of the brain, such as the frontal cortex, multiple parts of the motor system, and basal ganglia, exhibit changes in beta power when various motor functions are performed (Kühn et al., 2004; Swann et al., 2009, 2011, 2012; Wessel, 2020; Wessel et al., 2016). Likewise, gamma rhythms can often be induced in visual areas (such as the primary visual cortex (V1)) by presenting specific visual stimuli such as gratings, and both the power and center frequency of the gamma bursts depend on the properties of the visual stimuli (Jia et al., 2013; Murty et al., 2018; Ray & Maunsell, 2015) as well as cognitive factors such as attention (Bosman et al., 2012; Chalk et al., 2010; Das & Ray, 2018; Ferro et al., 2021; Vinck et al., 2013).

The gamma rhythm is hypothesized to provide a mechanism for communication across brain areas (Fries, 2015) or provide a temporal reference for the spiking activity that codes information relative to the rhythm (Fries et al., 2007), for which the rhythm should be sustained for behaviorally relevant time scales. However, several studies have shown that beta and gamma oscillations occur as bursts of small durations of the order of 100–200 ms (S. Burns et al., 2011; Feingold et al., 2015; Lundqvist, 2016; Rols et al., 2001; Xing et al., 2012). Some studies have suggested that gamma bursts spontaneously arise with matched timing and frequency to facilitate information flow on a cycle-by-cycle basis (Palmigiano et al., 2017). In some cases, even sustained rhythms may appear bursty in noisy data (van Ede et al., 2018).

Traditional methods for detecting bursts include spectral methods which use the time–frequency power spectrum of the signal computed using methods such as the Continuous Gabor Transform (CGT) (S. P. Burns et al., 2010; Xing et al., 2012) or Wavelet Transform (Rols et al., 2001; Roux et al., 2007). In these methods, bursts are detected as epochs in the time–frequency plane where the power (in the gamma frequency range) exceeds a particular threshold. In addition, some constraints on phase consistency may be imposed (S. P. Burns et al., 2010). Recently, a spectral estimator has been proposed that uses a set of wavelets (superlets) with increasingly constrained bandwidths that are geometrically combined in order to maintain good temporal resolution of single wavelets and gain frequency resolution in upper bands (Moca et al., 2021). However, these spectral estimation-based methods have a fundamental limitation since, in many cases, the spectral estimator follows an exponential distribution (Brillinger, 1972; Jarvis & Mitra, 2001), due to which the estimated power is low most of the time, with occasional time points with high power. This causes even sustained oscillations to appear bursty (Subhash et al., 2018). Another class of methods uses filtering-based techniques, where the signal is bandpass filtered in the frequency range of interest and smoothed with a window of suitable length. Then, the burst onset and duration are estimated by finding the time points where the estimated amplitude exceeds a particular threshold (Feingold et al., 2015). Another method used the Hilbert transform to estimate the instantaneous power (Lundqvist, 2016). Although these methods do not involve direct spectral estimation, the power estimation and thresholding step are mathematically equivalent to spectral estimation, which makes the output of power-dependent estimation to appear fundamentally bursty in nature.

We have previously shown that in data with synthetically injected gamma bursts of known lengths, traditional methods failed to pick up long gamma bursts, especially when the gamma power is low (Subhash et al., 2018). We then proposed a method based on the Matching Pursuit (MP) algorithm, which finds bursts completely in the time domain and is able to pick up longer bursts in synthetic data. MP decomposes a signal into a linear combination of functions (called atoms) taken iteratively from a large, overcomplete dictionary and identifies bursts based on the parameters of the chosen atoms. We found that MP-based burst estimation was much more robust to the choice of threshold. In real data recorded from two monkeys that passively viewed achromatic gratings, the median gamma burst lengths were about 300 ms (three times longer than traditional methods), although the mode gamma burst length was 100 ms, suggesting that gamma oscillations tend to occur in short bursts with occasional long bouts (Subhash et al., 2018). Similar results were obtained for the gamma oscillations induced by hue patches (Krishnakumaran & Ray, 2023).

In addition to burst detection, MP-based decomposition of neurophysiological data offers several advantages. First, brain signals such as local field potentials (LFP) often have transient responses related to stimulus onset, eye movements, or an action potential, which are not well described using sinusoids but instead resemble a Gaussian (see Krishnakumaran & Ray, 2023; Ray & Maunsell, 2015 for a detailed discussion). In particular, spikes are associated with a typical “spike-related-transient” in the LFP that can be well approximated by a negative Gaussian with a width of a few milliseconds (see Ray et al., 2008, figure 2 for MP-based decomposition of the spike-triggered-average of the LFP). This Gaussian has energy over a broad frequency range. Its energy is masked out by the “1/f” noise at lower frequencies but is visible at frequencies in the high-gamma range (>80 Hz). Since MP uses such Gaussians as basis functions, we found that the MP-based time–frequency spectrogram accurately tracked the multiunit firing rate in the high-gamma range (see Ray & Maunsell, 2011; Ray et al., 2008 for more details).

Despite these advantages over traditional methods, MP has some limitations. While convergence of MP is guaranteed, it is asymptotic, which means that for a finite-sized dictionary and a finite number of iterations, the residual error still may be large (Mallat & Zhang, 1993). Further, the residue (difference between the original signal and the reconstructed signal from the selected atoms) at each iteration is only orthogonal to the previously selected atom and may be correlated with other selected atoms, which means that residue can still be represented in terms of previously selected atoms (Mallat & Zhang, 1993). One way to address this issue is to make the residue orthogonal to all previously selected atoms, which is achieved using Orthogonal Matching pursuit (OMP). The residual error in OMP converges faster than MP, and it has been shown to provide better performance in high-precision applications (Pati et al., 1993). Both MP and OMP perform well if the dictionary has a large number of atoms, so that a small subset of the atoms has a high correlation with the signal to be decomposed. Hence, their performance improves with the dictionary size. However, when a large dictionary size is used, these algorithms are very slow. Recently, Canolty and Womelsdorf (2018) proposed a Multiscale Adaptive Gabor Expansion (MAGE) algorithm where a parameter reassignment step is used to refine the selected atom, which they used along with MP to estimate bursts.

In this paper, we present a Matlab-based toolbox called Burst estimation using Atomic decomposition (BEAD), which implements four algorithms: MP, OMP, OMP-MAGE, and a new method called OMP-GEAR, which performs parameter reassignment using a different approach as compared with OMP-MAGE. In addition, this toolbox implements the traditional methods discussed above. While MP (Subhash et al., 2018) and MP-MAGE (Canolty & Womelsdorf, 2018) have been implemented previously, OMP, OMP-MAGE, and OMP-GEAR are implemented for the first time for gamma burst duration estimation. Methods to generate synthetic data containing gamma bursts of known durations are included. Raw data from the two monkeys used in Subhash et al. (2018) are also provided. This comprehensive toolbox will allow researchers to test various burst estimation methods on synthetic and real data and quantify their respective complexity–performance tradeoffs.

## 2 Materials and Methods

### 2.1 Dataset description

The dataset used in the study (Subhash et al., 2018) consists of the Local Field Potential (LFP) collected from two adult female bonnet monkeys (macaca radiata), aged 13 and 17 years. The LFP signals were recorded by a 10 × 10 microelectrode array (96 active platinum electrodes; Utah array; Blackrock Microsystems) surgically implanted under general anesthesia. The Utah array was placed in each monkey’s primary visual cortex (area V1) of the right cerebral hemisphere (approximately 15 mm from the occipital ridge and 15 mm laterally from the midline). The receptive fields of neurons recorded from the microelectrodes were centered in the lower left quadrant of the visual field with eccentricities between 1.3 and 4.5 degrees.

The LFP signals were recorded while the monkeys performed a reward-based task where they fixated on a small spot in the center of the LED screen (BenQ XL2411, 1280 × 720 resolution, 100 Hz Refresh rate, linearized for contrast), while a large static grating of radius 4.8 degrees covering all the receptive fields was shown on screen. These grating were shown at five contrast levels: 0, 25, 50, 75, 100.

### 2.2 Synthetic LFP model

Synthetic LFP is generated by injecting a random number of Gabor atoms (Subhash et al., 2018) of the required duration (burst length) into the spontaneous LFP (denoted by ϵ). The spontaneous LFP is the signal, ϵ, recorded from the monkey brain when no stimulus (a blank screen) is shown to it. The spontaneous LFP has a sampling frequency of 250 Hz and an observation time of ≈4 s, so that the signal length is L≈1000 ; it acts as additive noise. The model is given by

$$f=K\left[\sum_{i=1}^{B}\alpha_{i}g_{\gamma_{i}}\right]+\epsilon ,$$ (1)

$$g_{\gamma }\left(t\right)=\frac{1}{\sqrt{s}}g\left(\frac{t-u}{s}\right)e^{i\xi t},$$ (2)

where $g_{\gamma_{i}}$ is a Gabor atom (the construction of $g_{\gamma_{i}}$ is explained in Section 2.3.1) with parameters $\gamma_{i}=(s_{i},u_{i},\xi_{i})$ . The number of injected bursts, denoted by B, is drawn from a Poisson distribution with a mean equal to the ratio of stimulus period duration to the burst length injected. Thus, the average number of bursts injected is inversely proportional to the burst length. The value of $u_{i}$ is sampled from a uniform distribution over the range of the burst time. If any pair of bursts overlaps in time, one of them is deleted. The value of $s_{i}$ is set as $s_{i}=\frac{\text{burst length}}{4}$. Note that, for Gabor atoms, $s_{i}$ denotes the standard deviation of the Gaussian window, and the burst duration is well approximated by four times the standard deviation. Hence, this choice of $s_{i}$ results in bursts of the desired length. The value of $\xi_{i}$ is sampled from a uniform distribution in gamma range (40–60 Hz). The value of $\alpha_{i}$ is sampled from a normal distribution with a certain mean and standard deviation. The mean is set equal to the difference in magnitude of the average FFT of the stimulus and the standard deviation of the spontaneous LFP standard deviation and the standard deviation is set to 10 percent of the difference. Finally, the burst signal $\left(\left[\sum_{i=1}^{B}\alpha_{i}g_{\gamma_{i}}\right]\right)$ is scaled with a constant, K, such that the spectrum of the overall synthetic LFP matches that of the real LFP in the gamma range.

### 2.3 Burst duration estimation using pursuit algorithms

The procedure of burst estimation using pursuit algorithms attempts to solve the sparse approximation problem: $\underset{x}{\text{min}}∥x{∥}_{0}$ subject to f=Dx . This problem is NP-hard and is computationally infeasible even for moderate dictionary sizes. Pursuit algorithms such as MP and OMP solve this problem suboptimally, and is explained below. First, we explain the construction of the dictionary, D.

#### 2.3.1 Dictionary

All the sparse recovery algorithms require a dictionary to work. Before discussing the algorithms, we discuss how the dictionary is formed. Dictionaries constructed using orthogonal bases such as the Fourier basis provide a poor representation of functions well localized in time, while wavelet bases are not well suited to represent functions whose Fourier transform has narrow high-frequency support (Mallat & Zhang, 1993). Therefore, dictionaries with waveforms well localized in both time and frequency are needed to represent nonstationary signals. Functions well localized in both time and frequency are called time–frequency atoms. A general family of time–frequency atoms can be generated by scaling, translating, and modulating a single Gaussian window function g(t) satisfying the constraint ∥g(t)∥ =1 . These time–frequency atoms can be parameterized by γ=(s,u,ξ), where s,u,ξ represent the scale, position, and frequency parameters, respectively. For a given $\gamma \in \Gamma ={\mathbb{R}}^{+}\times {\mathbb{R}}^{2}$, recall that $g_{\gamma }\left(t\right)$ can be written as (2). A dictionary containing a large number of atoms is constructed by gridding the parameter space Γ, and selecting an atom in that grid. We note that the parameter γ completely specifies the atom. From practical considerations, we limit the range of values of the scale, position, and frequency (s,u,ξ) parameters as s∈[1,L], u∈[0,L] and $\xi \in \left[0,\frac{F_{s}}{2}\right]$, where L is the observation duration of the LFP signal f in seconds and $F_{s}$ is the sampling frequency of f. The dictionary is constructed by dividing the parameter space Γ into a grid, and, if required, subsampling the grid to obtain the desired number of atoms.

##### 2.3.1.1 Dyadic dictionary

The most common approach is to form a grid in the dyadic scale, so that

$\gamma =(2^{j},p2^{j}\Delta u,\;k2^{-j}\Delta \xi )$ , $\Delta u=\frac{1}{2},\;\Delta \xi =\frac{F_{s}}{2}$, and j,k, and p are integers spanning $0<j<{\text{log}}_{2}\;Z$ , $0\leq p<Z2^{j+1}$ and $0\leq k<2^{j+1}$ , where $Z=LF_{s}$. A dyadic dictionary has O(Z log Z) atoms (Mallat & Zhang, 1993).

##### 2.3.1.2 Stochastic dictionary

Using a dyadic dictionary introduces a bias because the scale parameter s is a power of 2 (Durka et al., 2001). An alternative approach is to form a finely spaced uniform grid and sample the required number of atoms uniformly at random from this dictionary. This results in the so-called stochastic dictionary (Durka et al., 2001). We have empirically observed that, on average, a stochastic dictionary produces better results than a dyadic dictionary.

#### 2.3.2 Matching pursuit (MP)

Given a signal f, dictionary D, and sparsity level M, the MP algorithm (Mallat & Zhang, 1993) produces the following sparse approximation after M iterations:

$$f=\sum_{n=0}^{M-1}\langle R^{\left(n\right)}\;f,g_{\gamma_{n}}\rangle g_{\gamma_{n}}+R^{\left(M\right)}\;f.$$ (3)

At each iteration, MP selects the atom $g_{\gamma_{n}}\in D$ with maximum magnitude of correlation with the residue:

$$g_{\gamma_{n}}=\underset{g_{\gamma }\in D}{\text{arg max}}\;\;\left|\langle g_{\gamma },R^{\left(n\right)}\;f\rangle \;\right|.$$ (4)

The residue in the first iteration is set as the signal itself. At each subsequent iteration, the residue is decomposed recursively: $R^{\left(n\right)}\;f=\langle R^{\left(n\right)}\;f,g_{\gamma_{n}}\rangle g_{\gamma_{n}}+R^{\left(n+1\right)}\;f$ . Due to (4), $R^{\left(n+1\right)}\;f$ is orthogonal to $g_{\gamma_{n}}$ but not all previously selected atoms. Hence, the residue is correlated to the previous n−1 atoms and can be represented in terms of these atoms also. Thus, an atom can be selected multiple times. As a consequence, MP does not make the best use of the selected atoms, and its convergence is only asymptotic.

#### 2.3.3 Orthogonal matching pursuit

OMP (Pati et al., 1993) is also an iterative algorithm that selects an atom at each iteration with the largest magnitude correlation with the residue. To obtain the best approximation of the signal with the selected atoms, at the kth iteration, OMP constructs an (L×k)-sized submatrix $D_{k}$ of the (L×N)-sized original dictionary D formed using the k atoms selected till iteration k, where L is the length of the signal to be decomposed into its atoms and N is the total number of atoms in the dictionary. The vector $x^{\left(k\right)}$ containing coefficients corresponding to the selected atoms is computed by solving the least-squares problem

$$x^{\left(k\right)}=\underset{x\in {\mathbb{R}}^{k}}{\text{argmin}}∥D_{k}x-f{∥}^{2}.$$ (5)

The solution to the above problem is $x^{\left(k\right)}={\left(D_{k}^{T}D_{k}\right)}^{-1}$ $D_{k}^{T}\;f$ , and the residue is updated as $R^{\left(k\right)}\;f=f-D_{k}x^{\left(k\right)}$ . This makes the residue orthogonal to all the previously selected atoms, and we get the least mean squared error approximation of the signal with the selected atoms. Note that $x^{\left(k\right)}$ contains the coefficients of the N-length vector x corresponding to the k atoms selected till the kth iteration of OMP. OMP converges in a finite number of iterations; in fact, it converges in at most m iterations, where m is the length of the signal (Pati et al., 1993).

##### 2.3.3.1 Phase correction

To properly account for the phase of the real-valued signal, we must construct two dictionaries: one with the Gabor atom formed using the cosine function and the other with Gabor formed using the sine function. Consider a signal $f=g(t)\;\text{cos}(\xi_{m}t+\phi )+\eta$ , where η is white Gaussian noise, m=1 or 2 are two potential frequencies. Suppose we have two dictionaries with two atoms each, $\text{Dcos}=[g(t)\;\text{cos}(\xi_{1}t),\;g(t)\;\text{cos}(\xi_{2}t)]$ and $\text{Dsin}=[g(t)\;\text{sin}(\xi_{1}t),\;g(t)\;\text{sin}(\xi_{2}t)]$ . Then, detecting the correct frequency is equivalent to non-coherent detection of frequency. It can be shown that when ϕ∼Unif [0, 2π] , the minimum probability of error is achieved when we choose the frequency $\xi_{m}$ such that

$$m=\underset{i=1,2}{\text{arg max}}|\langle f,g\left(t\right)\;\text{cos}\left(\xi_{i}t\right)\rangle {|}^{2}\;+|\langle f,g\left(t\right)\;\text{sin}\left(\xi_{i}t\right)\rangle {|}^{2}\;.$$ (6)

The phase of the signal is then estimated as

$$\phi^{*}=\text{arctan}\left(\frac{\langle f,g(t)\;\text{sin}(\xi_{m}t)\rangle }{\langle f,g(t)\;\text{cos}(\xi_{m}t)\rangle }\right).$$ (7)

This phase estimate is the maximum likelihood estimate if the noise η is uncorrelated with the selected Gabor atom.

To adapt the OMP algorithm for real signals with unknown phase, we must thus start with two dictionaries containing the signals $g_{\gamma_{ic}}\left(t\right)=\frac{1}{\sqrt{s_{i}}}g\left(\frac{t-u_{i}}{s_{i}}\right)\text{ cos}\left(\xi_{i}t\right)$ and $g_{\gamma_{is}}\left(t\right)=\frac{1}{\sqrt{s_{i}}}g\left(\frac{t-u_{i}}{s_{i}}\right)\text{ sin}\left(\xi_{i}t\right)$. We normalize these functions to have unit norm while forming the dictionary. At iteration k, we select the parameter $\gamma_{k}$ using the residue $R^{\left(k-1\right)}$ from the previous iteration such that

$$k=\underset{i}{\text{arg max}}|\langle R^{\left(k-1\right)},g_{\gamma_{ic}}\rangle {|}^{2}+\;|\langle R^{\left(k-1\right)},g_{\gamma_{is}}\rangle {|}^{2},$$ (8)

and the phase is estimated as

$$\phi_{k}=\text{arctan}\left(\frac{\langle R^{\left(k-1\right)},g_{\gamma_{ks}}\rangle }{\langle R^{\left(k-1\right)},g_{\gamma_{kc}}\rangle }\right).$$ (9)

These two steps are necessary to represent signals using real-valued Gabor atoms.

#### 2.3.4 OMP-MAGE

MP and OMP select the atom with the largest correlation, but the selected atom is typically not the true atom (depending on the dictionary, it may be close to the true atom). This is because the parameter space used to represent the atoms is continuous valued, and we sample this space to construct the atoms (columns) of the dictionary. Hence, the dictionary does not contain all possible atoms. Multiscale adaptive Gabor expansion (MAGE) (Canolty & Womelsdorf, 2018) is a method in which the atom detected at each iteration is treated as an initial estimate. This estimate is further refined to move closer to the true atom. In MAGE, an atom is rewritten as

$$g_{k}\left(t\right)=\sqrt[{4}]{2}\text{exp}\left(-\frac{s_{k}}{4}-\pi {\left(t-u_{k}\right)}^{2}e^{-s_{k}}+2\pi i\xi_{k}(t-u_{k})\right),$$ (10)

which is mathematically equivalent to (2). If we set $s_{k}=2\;{\text{log}}_{e}\;s$ , the mapping is one-to-one, so for every s, there is a corresponding $s_{k}$. The approach used in MAGE is one of curve fitting: the atom selected $g_{p}$ is refined to a new atom $g_{t}$ such that $g_{t}$ fits the residue better at that iteration. Since $g_{t}$ has three degrees of freedom s,u, and ξ, we need three equations to update it. We explain the update equations below.

An analytical expression for the inner product between two atoms is given by

$$\langle g_{t},g_{p}\rangle =A_{\rho \;}\text{exp}[i\theta_{\rho }],$$ (11)

where

$$A_{\rho }=\sqrt{\text{sech}\left[\frac{s_{\Delta }}{2}\right]}\text{exp}\left[-\frac{\pi e^{-s_{p}}u_{\Delta }^{2}}{1+e^{s_{\Delta }}}-\frac{\pi e^{s_{p}}\xi_{\Delta }^{2}}{1+e^{-s_{\Delta }}}\right].$$ (12)

$$\theta_{\rho }=-\frac{2\pi u_{\Delta }\xi_{\Delta }}{1+e^{-s_{\Delta }}}-2\pi \xi_{p}u_{\Delta }$$ (13)

and $u_{\Delta }=u_{t}-u_{p},\xi_{\Delta }=\xi_{t}-\xi_{p},s_{\Delta }=s_{t}-s_{p}$. Using the chain rule, the derivative of the inner product with respect to a parameter $x_{p}$ can be written as

$$\frac{\partial \langle g_{t},g_{p}\rangle }{\partial x_{p}}=\zeta_{x_{p}}\langle g_{t},g_{p}\rangle .$$ (14)

We can calculate $\frac{\partial \langle g_{t},g_{p}\rangle }{\partial x_{p}}$ for all the three parameters $s_{p},u_{p},\xi_{p}$ and $\langle g_{t},g_{p}\rangle$ from the data. Also, we can obtain an analytical expression for $\zeta_{x_{p}}$ by differentiating (11) with respect to $s_{p},u_{p},\xi_{p}$. By equating the analytical expression to the value calculated from the data, we arrive at the following closed-form analytical expressions for the parameters of the new atom:

$$s_{t}^{*}=s_{p}+\text{log}\left[\frac{2\pi e^{s_{p}}}{e^{s_{p}}(e^{s_{p}}ℛ{\left[\zeta_{u_{p}}\right]}^{2}-4\pi ℛ[\zeta_{s_{p}}]+\pi )-ℛ{\left[\zeta_{\xi_{p}}\right]}^{2}}-1\right]$$ (15)

$$\xi_{t}^{*}=\xi_{p}+\frac{\left(e^{-s_{p}}+e^{-s_{t}^{*}})ℛ[\zeta_{\xi_{p}}\right]}{2\pi }$$ (16)

$$u_{t}^{*}=u_{p}+\frac{\left(e^{s_{p}}+e^{s_{t}^{*}})ℛ[\zeta_{u_{p}}\right]}{2\pi }.$$ (17)

The above update equations are used as follows. At the nth iteration of the algorithm, suppose $g_{\gamma_{n,0}}$ is the estimate of an atom that we get from MP or OMP using the residue computed at the start of that iteration, and suppose the true atom to be found (e.g., the atom present in the signal that is closest to $g_{\gamma_{n,0}}$ ) be $g_{\gamma_{n}}$. We can write the dot product between the residue at iteration n and the atom $g_{\gamma_{n}}$ as

$$\langle R^{n}f,g_{\gamma_{n}}\rangle =\langle R^{n}f,g_{\gamma_{n,0}}\rangle \langle g_{\gamma_{n,0}},g_{\gamma_{n}}\rangle +\langle R^{n+1}f,g_{\gamma_{n}}\rangle .$$ (18)

If $g_{\gamma_{n,0}}$ coincides with $g_{\gamma_{n}}$, the interference term $\langle R^{n+1}f,g_{\gamma_{n}}\rangle =0$ . Using this, MAGE reassignment proceeds by neglecting the interference term and using (18) to reassign the atom parameters. This is further explained in Section 2.3.5.

##### 2.3.4.1 OMP-MAGE versus MAGE

The original MAGE algorithm (Canolty & Womelsdorf, 2018) was based on the use of MP to find the initial atom, whereas, in this work, we propose to use OMP to find the initial atom(s). Empirically, it is known that OMP has a better convergence rate than MP (Pati et al., 1993); see also Section 4. However, the use of MAGE in conjunction with OMP instead of MP requires us to form the dictionary differently. Specifically, we form one dictionary with the cosine function, $g_{kc}\left(t\right)=$ $\sqrt[{4}]{2\;}\text{exp}\left(-\frac{s_{k}}{4}-\pi {\left(t-u_{k}\right)}^{2}e^{-s_{k}}\right)\text{cos}\left(2\pi i\xi_{k}(t-u_{k})\right)$ and the other with the sine function $g_{ks}\left(t\right)=\sqrt[{4}]{2\;}\text{exp}(-\frac{s_{k}}{4}-\pi (t-$ $u_{k}{)}^{2}e^{-s_{k}})\text{sin}(2\pi i\xi_{k}(t-u_{k}))$ . This allows us to use the real-valued update Equations (15)–(17) to perform atom reassignment.

#### 2.3.5 Gabor expansion with atom reassignment (GEAR)

As discussed above, MAGE (Canolty & Womelsdorf, 2018) finds a new atom $g_{t}$ that is best matched with the residue at the current iteration by differentiating the inner product between the residue and the initial atom estimate rather than directly solving for the atom parameters. In effect, MAGE relies on a first-order Taylor series approximation of the atoms around the selected atom, and the resulting approximation error (see the note after (18)) can result in suboptimal parameter estimates. We now present a novel alternative approach, where, instead of using a first-order Taylor approximation, we directly fit the residue to a new atom, $g_{t}$, using the magnitude of the inner product between the residue and the initial atom, $g_{p}$, found by OMP. The magnitude of the inner product between two Gabor atoms is given by

$$\langle g_{t},g_{p}\rangle =\sqrt{\text{sech }\left[\frac{s_{\Delta }}{2}\right]}\text{exp }\left[-\frac{\pi e^{-s_{p}}u_{\Delta }^{2}}{1+e^{s_{\Delta }}}-\frac{\pi e^{s_{p}}\xi_{\Delta }^{2}}{1+e^{-s_{\Delta }}}\right].$$ (19)

Suppose we want to fit the residue R at any iteration to a Gabor atom $g_{t}$ and we have an initial estimate $g_{p}$ with parameters $\left(s_{p},\xi_{p},u_{p}\right)$ . We take another atom $g_{p1}$ with parameters $(s_{p1},\xi_{p1},u_{p1}),$ where $u_{p1}=u_{p}+\Delta u$ and $\xi_{p1}=\xi_{p}+\Delta \xi$ . Since we wish to fit a true atom $g_{t}$ to the residue, we take the ratio of the magnitude of the inner products of R with $g_{p}$ and $g_{p1}$ , and simplify to get

$$\begin{array}{cc} \begin{array}{c} b\left[\left(\xi_{p1}^{2}-\xi_{p}^{2})-e^{-2s_{p}}(u_{p1}^{2}-u_{p}^{2}\right)\right]+2e^{-2s_{p}}(\pi e^{s_{p}}-b)\;u_{t}(u_{p}-u_{p1})+2b\xi_{t}[\xi_{p}-\xi_{p1}]\\ \;\;\;\;\;\;\;\;\;\;\;\;\;\;\;\;\;\;\;\;\;\;\;\;\;\;\;\;\;\;\;\;\;\;=\text{log}\left[\frac{\left|\langle R,g_{p}\rangle \right|}{\left|\langle R,g_{p1}\rangle \right|}\right]-\;\pi e^{-s_{p}}(u_{p1}^{2}-u_{p}^{2}), \end{array} & \end{array}$$ (20)

where $b=-\frac{\pi e^{s_{p}}\Delta u^{2}}{1+e^{-\Delta s}}$ . Defining intermediate variables $B_{u}=2e^{-2s_{p}}(\pi e^{s_{p}}-b)u_{t}$ and $B_{\xi }=2b\xi_{t}$, we get a linear equation in three variables $b,B_{u},B_{\xi }$:

$$\begin{array}{cc} \begin{array}{c} b\left[(\xi_{p1}^{2}-\xi_{p}^{2})-e^{-2s_{p}}(u_{p1}^{2}-u_{p}^{2})]+B_{u}(u_{p}-u_{p1})+B_{\xi }[\xi_{p}-\xi_{p1}\right]\\ =log\left[\frac{\left|\langle R,g_{p}\rangle \right|}{\left|\langle R,g_{p1}\rangle \right|}\right]-\;\pi e^{-s_{p}}(u_{p1}^{2}-u_{p}^{2}). \end{array} & \end{array}$$ (21)

The right-hand side of (21) can be calculated from residue, initial estimate, and test atom $g_{p1}$ . Since the left-hand side has three unknowns, we need three equations. This can be obtained by taking two more testing atoms $g_{p-1}$ with parameters $\left(s_{p},u_{p-1},\xi_{p-1}\right)$ and $g_{p1-1}$ with parameters $\left(s_{p},u_{p1},\xi_{p-1}\right)$ , $u_{p-1}=u_{p}-\Delta u$ and $\xi_{p-1}=\xi_{p}-\Delta \xi$ :

$$\begin{array}{l} \left[\begin{array}{ccc} \left(\xi_{p1}^{2}-\xi_{p}^{2})-e^{-2s_{p}}(u_{p1}^{2}-u_{p}^{2}\right) & \left(u_{p}-u_{p1}\right) & \left(\xi_{p}-\xi_{p1}\right)\\ \left(\xi_{p-1}^{2}-\xi_{p}^{2})-e^{-2s_{p}}(u_{p-1}^{2}-u_{p}^{2}\right) & \left(u_{p}-u_{p-1}\right) & \left(\xi_{p}-\xi_{p-1}\right)\\ \left(\xi_{p-1}^{2}-\xi_{9}^{2})-e^{-2s_{p}}(u_{p1}^{2}-u_{p}^{2}\right) & \left(u_{p}-u_{p1}\right) & \left(\xi_{p}-\xi_{p-1}\right) \end{array}\right]\left[\begin{array}{c} b\\ B_{u}\\ B_{\xi } \end{array}\right]\\ \;\;\;\;\;\;\;\;\;\;\;\;\;\;\;\;\;\;\;\;\;\;\;\;\;\;\;\;\;\;=\left[\begin{array}{c} \log\left[\frac{\left|\langle R,g_{p}\rangle \right|}{\left|\langle R,g_{p-1}\rangle \right|}\right]-\pi e^{-s_{p}}(u_{p1}^{2}-u_{p}^{2})\\ \log\left[\frac{\left|\langle R,g_{p}\rangle \right|}{\left|\langle R,g_{p-1}\rangle \right|}\right]-\pi e^{-s_{p}}(u_{p-1}^{2}-u_{p}^{2})\\ \log\left[\frac{\left|\langle R,g_{p}\rangle \right|}{\left|\langle R,g_{p1,-1}\rangle \right|}\right]-\pi e^{-s_{p}}(u_{p1}^{2}-u_{p}^{2}) \end{array}\right]. \end{array}$$ (22)

Using the system of equations above, we solve for the three variables $b,B_{u},B_{\xi }$. Then, we can reassign the atom parameter values as

$$s_{t}=s_{p}-\text{log}\left[\frac{\pi }{b}e^{s_{p}}-1\right]$$ (23)

$$u_{t}=\frac{B_{u}}{2e^{-2s_{p}}(\pi e^{s_{p}}-b)}$$ (24)

$$\xi_{t}=\frac{B_{\xi }}{2b}.$$ (25)

Note that, in order to obtain the system of equations in (22), we perturb only time and frequency parameters, that is, we skip taking a finite difference with respect to the scale parameter. This is a pragmatic choice, as it allows us to obtain the above closed-form expressions for the atom reassignment. The parameters Δu and Δξ must be chosen to be of a small value for the algorithm to converge; we use $\Delta u=\frac{0.1}{L}$ and $\Delta \xi =\frac{0.1}{F_{s}}$, where L is the number of samples in the signal and $F_{s}$ is the sampling rate. For signals with different parameters such as sampling rate and number of samples, the parameters Δu and Δξ can be tuned to get better results. We call this reassignment method used in conjunction with our implementation of OMP the OMP-GEAR algorithm.

From the sparse approximation of the signal using any of the three methods, we obtain a set of atoms, equivalently, a set of parameter tuples that can be used to represent the signal. These parameters are used to estimate burst duration (Subhash et al., 2018). Atoms with frequency in the gamma range (40–60 Hz) and position parameters in the stimulus period (0–2 s) are selected as candidate gamma bursts. Finally, atoms whose coefficients exceed a *threshold* are included in the final estimate of the bursts. The threshold is set as a fraction (called *threshold fraction* in this paper) of the mean of the largest coefficient obtained in the spontaneous period. The burst duration is set as four times the atom’s standard deviation, and the median of all these bursts across all trials is taken as the estimated burst duration. Burst durations exceeding 2 s are discarded as gamma bursts are typically more abrupt than the slow decay of a Gaussian window.

### 2.4 MP, OMP, and OMP-MAGE implementation

We use the implementation of MP by Durka et al. (2001), which uses a stochastic dictionary instead of a dyadic dictionary as used in the original implementation by Mallat and Zhang (1993). This implementation is entirely written in C, with various optimizations to reduce computational time, making MP much faster than OMP and OMP-MAGE.

Our implementation of OMP, outlined above, has three major differences from conventional implementations of the algorithm. First, the (large) dictionary is not constructed in advance; a stochastic dictionary is formed on the fly using the dictionary size and sampling rate as inputs, inspired by Durka et al. (2001). Second, unlike conventional OMP where the inner product is computed with all atoms in the dictionary, the atom with the highest magnitude correlation is selected, we form two Gabor dictionaries with the same parameters: one constructed using the cosine function and the other constructed using the sine function. Then we compute the inner product with all the atoms in the two dictionaries and choose the parameter that has the largest sum of squared of inner products of corresponding cosine and sine Gabor atoms. Third, we add a phase correction step by setting the phase of the sinusoid as the inverse tangent of the ratio of the inner products of the Gabor atom with sine and cosine functions. These enhancements are necessary for the correct representation of the signal.

### 2.5 Limitations of OMP and OMP-MAGE

The main limitation of OMP and OMP-MAGE is that these methods are slower than MP. There are two reasons for this: first, OMP and OMP-MAGE are both implemented in Matlab, whereas MP is implemented in C, making the latter faster. Second, the MP implementation computes and stores inner products between dictionary atoms in advance, which speeds up the algorithm. However, in Matlab, the memory requirement for such a pre-computation is too large for practical implementation. To elaborate, in MP, atoms are computed when needed and only the atom parameters are stored, which requires multiple nested loops when implemented in Matlab, which makes the Matlab implementation of the same algorithm much slower than the C implementation.

There are faster ways to implement OMP, for example, using the inverse Cholesky factorization. When implemented in C, this can make our method much faster. As we saw earlier, OMP-MAGE offers comparable performance as OMP even with a much smaller sized dictionary (about 10x smaller). Hence, with a better implementation, the computational time of OMP-MAGE can be reduced, making it computationally superior to the MP-based method.

### 2.6 Burst duration estimation using the Hilbert Transform

The analytical signal is formed from the given signal f as

$$A_{f}=f+jH\left[f\right],$$ (26)

where H denotes the Hilbert Transform operator. This signal is band pass filtered in the gamma frequency range (40–60 Hz), and the envelope is found by taking $|\;A_{f}\;{|}^{2}$. A burst is registered when the power exceeds three times the median power. The start and end of the burst are taken as the times when $|\;A_{f}\;{|}^{2}$ falls below 1.5 times its median power.

## 3 Results

The generation of synthetic data has been explained in detail in our previous study (Subhash et al., 2018); we briefly describe it in Section 2.2. We injected multiple non-overlapping gamma bursts of known durations in the spontaneous LFP data while ensuring that the total power in the gamma range was similar to the gamma power observed in real data. Each trial of the spontaneous LFP data was recorded at a sampling rate of 250 Hz for about 4 s duration from a monkey when a blank screen was displayed to it. When the gamma power was high, methods based on Hilbert Transform and Wavelet transform performed well, although the CGT-based method failed even in this condition (see Subhash et al., 2018, figure 3C). However, when the injected gamma bursts had low power, even Hilbert and Wavelet-based methods failed, and only the MP-based method was able to estimate the duration of long bursts properly (see Subhash et al., 2018, figure 3I).

**Fig. 1.**
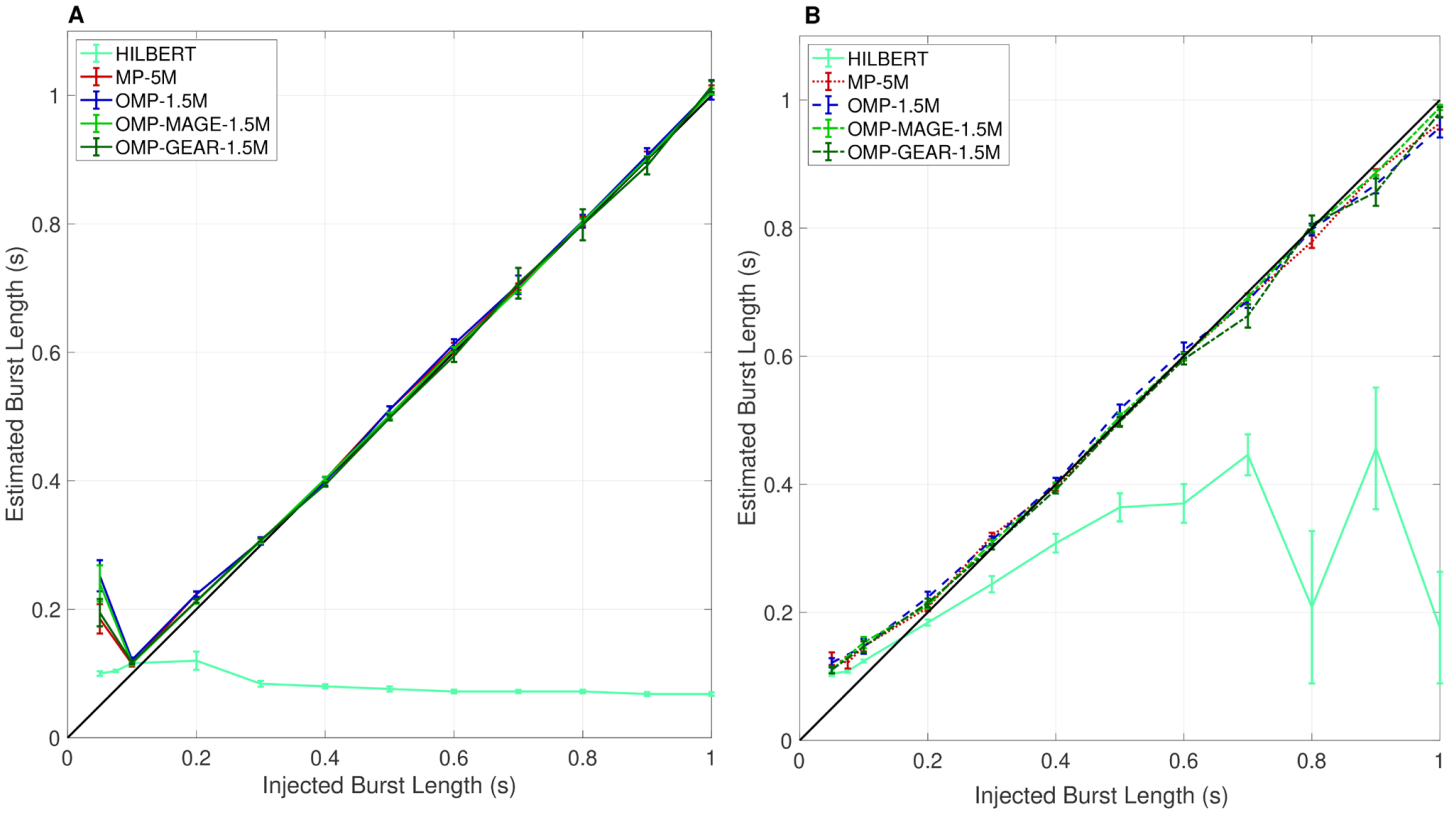
Comparison of gamma duration estimators. (A) Injected versus estimated burst lengths, when the injected burst has one-fourth the power of the real LFP in the gamma range. The bursts are non-overlapping, and the injected bursts are of duration 0.05 s, 0.075 s, and 0.1 to 1 s in steps of 0.1 s. The threshold fraction (see Section 2.3.5 for the definition) is set to 0.5. (B) Injected burst versus median burst lengths when the power of the injected burst matches that of the real LFP in the gamma range. Overlap of bursts is allowed, and the injected bursts are of duration 0.05 s, 0.075 s, and 0.1 to 1 s in steps of 0.1 s. The threshold fraction is set to 0.5.

Figure 1A shows the median of the estimated burst duration across 102 trials as a function of the injected burst lengths. All four atomic decomposition-based methods estimate the burst duration with high accuracy. For example, for an injected burst length of 300 ms, the estimated burst lengths were 306 ± 3.8 ms, 306 ± 5.7 ms, 306 ± 3.7 ms, and 308 ± 2.8 ms using MP with 5 million atoms (MP-5M), OMP with 1.5 million atoms (OMP-1.5M), OMP-MAGE with 1.5 million atoms (OMP-MAGE-1.5M), and OMP-GEAR with 1.5 million atoms (OMP-GEAR-1.5M), respectively. In contrast, the Hilbert-based method performed poorly, with an estimated burst duration of 84 ± 4.6 ms.

The only exception was when 50 ms bursts were injected. Because the total number of bursts was very high in this case (the number of bursts injected was increased in inverse proportion to the burst duration), two nearby bursts sometimes tended to get estimated as a single longer burst. Further, the sufficient conditions for sparse recovery are not met in this case (see Section 4.1), so it is not surprising that the atomic decomposition-based methods fail. However, the four atomic methods performed comparably in this condition, with estimated burst lengths of 185 ± 22.8 ms, 252 ± 24.2 ms, 240 ± 28.1 ms, and 195 ± 21.3 ms using MP with 5 million atoms (MP-5M), OMP with 1.5 million atoms (OMP-1.5M), OMP-MAGE with 1.5 million atoms (OMP-MAGE-1.5M), and OMP-GEAR with 1.5 million atoms (OMP-GEAR-1.5M), respectively. Although it appears that, in this case, the Hilbert method overestimated the burst length the least (at 100 ± 3 ms), the curve for Hilbert was almost horizontal, which means that it outputs the same burst duration for any injected burst duration.

To make burst estimation even more challenging, we removed the requirement that the bursts be non-overlapping. Figure 1B shows the results for this case. All four atomic methods continued to perform well, while the Hilbert-based method gave poor results.

**Fig. 2.**
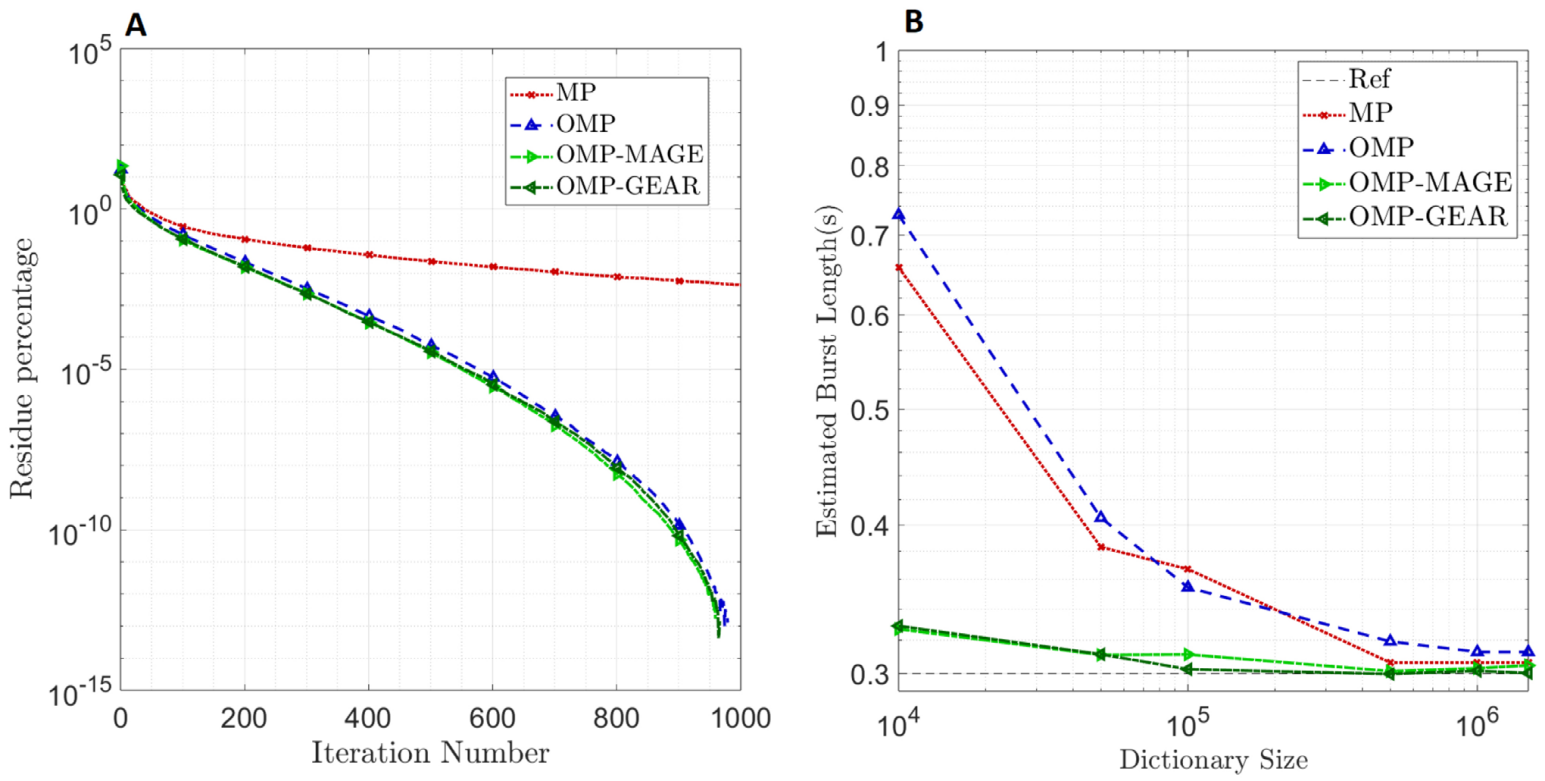
(A) Convergence analysis of methods: Residue energy (percentage relative to the signal energy) versus iteration number. (B) Performance of atomic methods as a function of the dictionary size: Estimated burst durations for the three methods for various dictionary sizes when bursts of duration 300 ms are injected (shown as a dashed line with label Ref).

In Figure 1, OMP, OMP-MAGE, and OMP-GEAR offer no improvement over MP, simply because we used a large dictionary (5 million atoms) and a large number of iterations, as determined in our previous study, to ensure that MP performed well for the synthetic data. However, such large dictionaries and iterations put severe constraints on the processing system and may not be desirable when memory or processing capacity needs to be minimized (e.g., for real-time applications). Therefore, we tested the performance of the three atomic methods as a function of the number of iterations and dictionary size. For this, we simulated synthetic LFP data and injected bursts of 300 ms. Figure 2A shows the percentage reduction in the energy of the residue (the as-yet unexplained portion of the signal) as a function of the number of iterations. To obtain the curves, a single trial was taken and the residue percentage was plotted for each method across 1000 iterations. Note that the curves are shown down to a residue percentage of $10^{-10}$ or lower simply to highlight the relative convergence behavior of the different algorithms; in practice, a residue percentage of $10^{-5}$ or so is sufficient to ensure that the useful features of the signal are extracted. As expected, OMP, OMP-MAGE, and OMP-GEAR converge much faster than MP, showing that if there is a constraint on the number of iterations, these methods can outperform MP. Figure 2B shows the estimated burst length as a function of the dictionary size. Here, 300 ms bursts were injected into the spontaneous LFP, and each point in the figure corresponds to the median of 102 trials. As the size of the dictionary reduces, OMP-MAGE and OMP-GEAR continue to perform well while MP and OMP significantly overestimate the burst duration. This illustrates the advantage of OMP-MAGE and OMP-GEAR relative to previous methods.

**Fig. 3.**
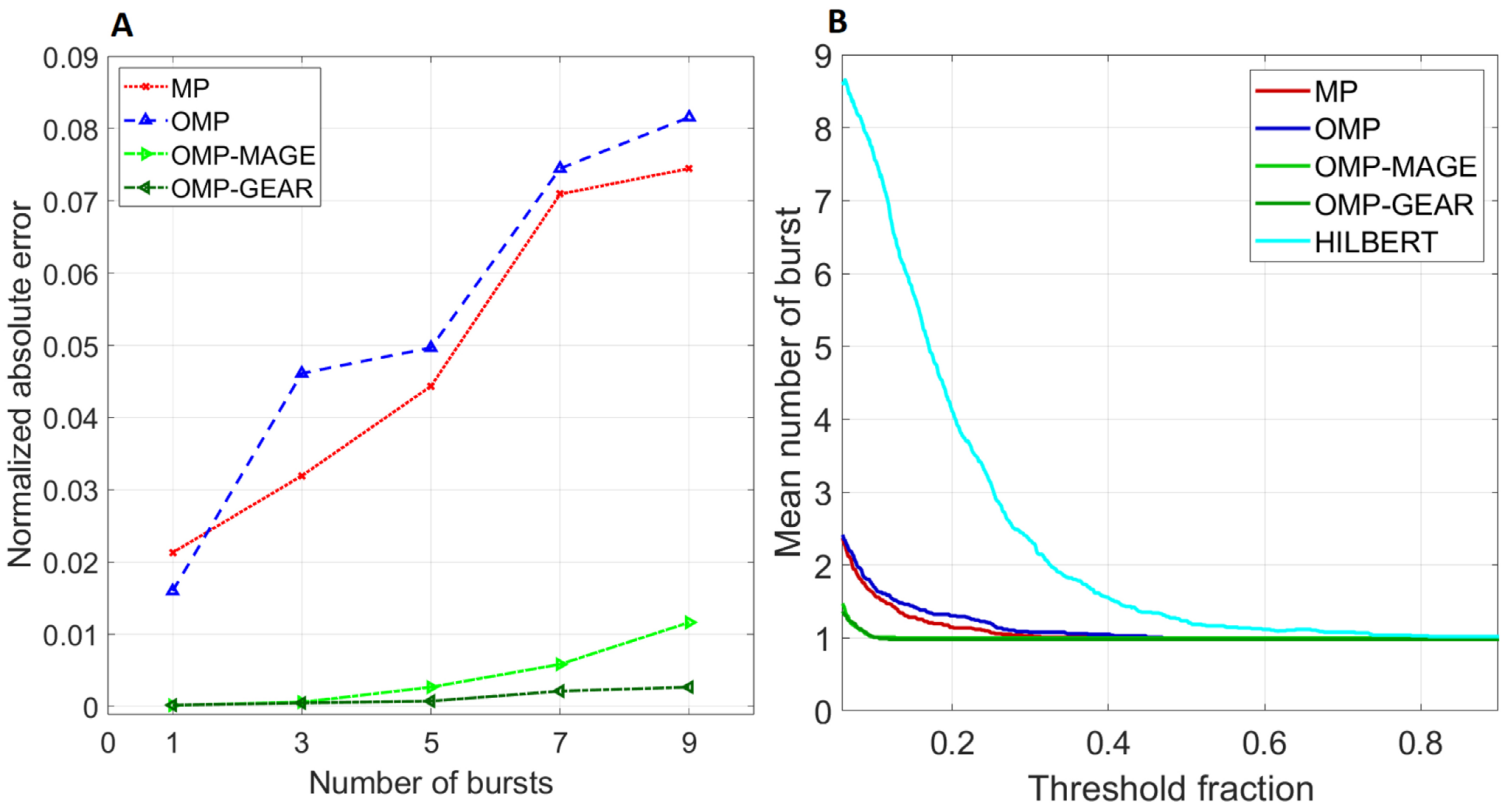
(A) Performance of atomic methods as a function of number of bursts injected: Normalized absolute error for increasing number of bursts when bursts of duration 300 ms are injected. (B) Performance of methods with varying threshold fractions: Mean number of bursts detected across 102 trials when the threshold fraction is varied between 0.06 and 0.9.

We tested the effect of increasing the extent of overlap between the bursts (see Fig. 3A). For this, we increased the number of overlapping bursts of 300 ms duration. To obtain each point in the graph, we injected a fixed number of 300 ms bursts, and found the median of the absolute error across 102 trials. We repeated this experiment six times, and plotted the average of the median absolute errors obtained from the six experiments. OMP-MAGE and OMP-GEAR were able to resolve multiple bursts better than MP and OMP. OMP-GEAR yielded lower error than OMP-MAGE when the number of bursts is high, showing that it is the best-performing algorithm. The new class of methods, namely, OMP-MAGE and OMP-GEAR, not only outperform MP and OMP, but also show an even greater resilience at low threshold fractions (see Fig. 3B). Here, we injected a single 300 ms burst and found the mean number of bursts detected across 102 trials for different values of the threshold fraction. The reason for this improved performance stems from the fact that, for classical methods, noise in the power estimate (either due to noise in the spectral estimator or in the data) leads to an abrupt termination of the burst, which is more pronounced when the threshold fraction is low. BEAD-based methods use the overall signal structure in the time domain to estimate the bursts, which makes them less sensitive to noise in the power/spectral domains.

**Fig. 4.**
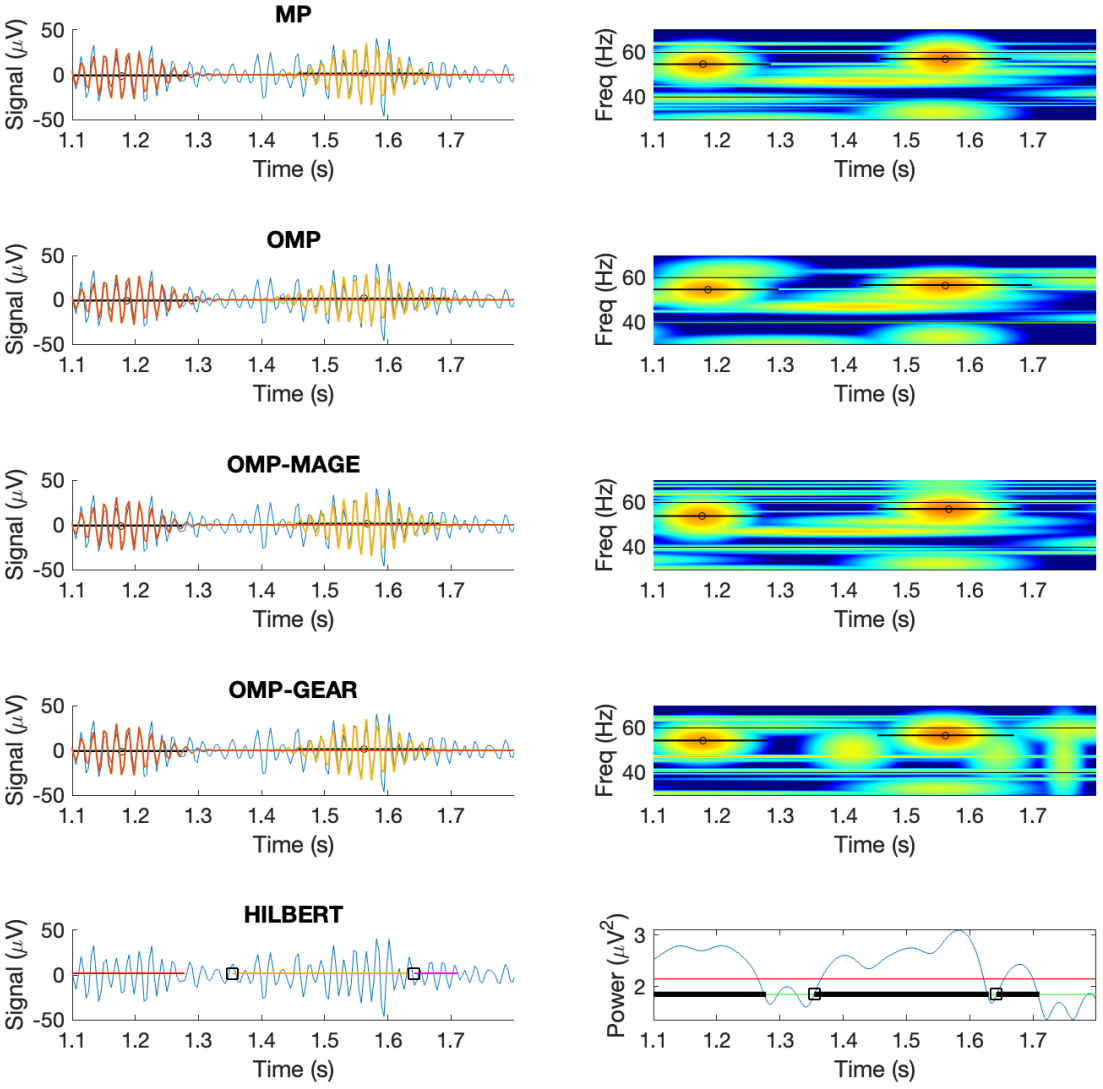
Visual illustration of the bursts detected by the different methods. Left: LFP overlaid with the detected atoms (LFP overlaid with the detected burst length for the Hilbert method); Right: Wigner–Ville time–frequency plots for atomic methods (power plot overlaid with detected burst length for the Hilbert method.)

To visually illustrate the bursts detected by the atomic methods and the Hilbert method, in the first column of Figure 4, we plot the LFP overlaid with detected atoms in case of atomic methods, and LFP overlaid with detected burst length in case of the Hilbert method. In the second column, we show Wigner–Ville time–frequency plots for atomic methods and the power plot overlaid with detected burst length for the Hilbert method. For this plot, we used a threshold fraction of 0.75 and a dictionary size of 2.5 M for MP and 1.5 M for OMP, OMP-MAGE, and OMP-GEAR. We see that all the atomic methods effectively capture the shape of gamma oscillation, while the Hilbert method tends to approximate a single large gamma oscillation with multiple smaller gamma oscillations.

**Fig. 5.**
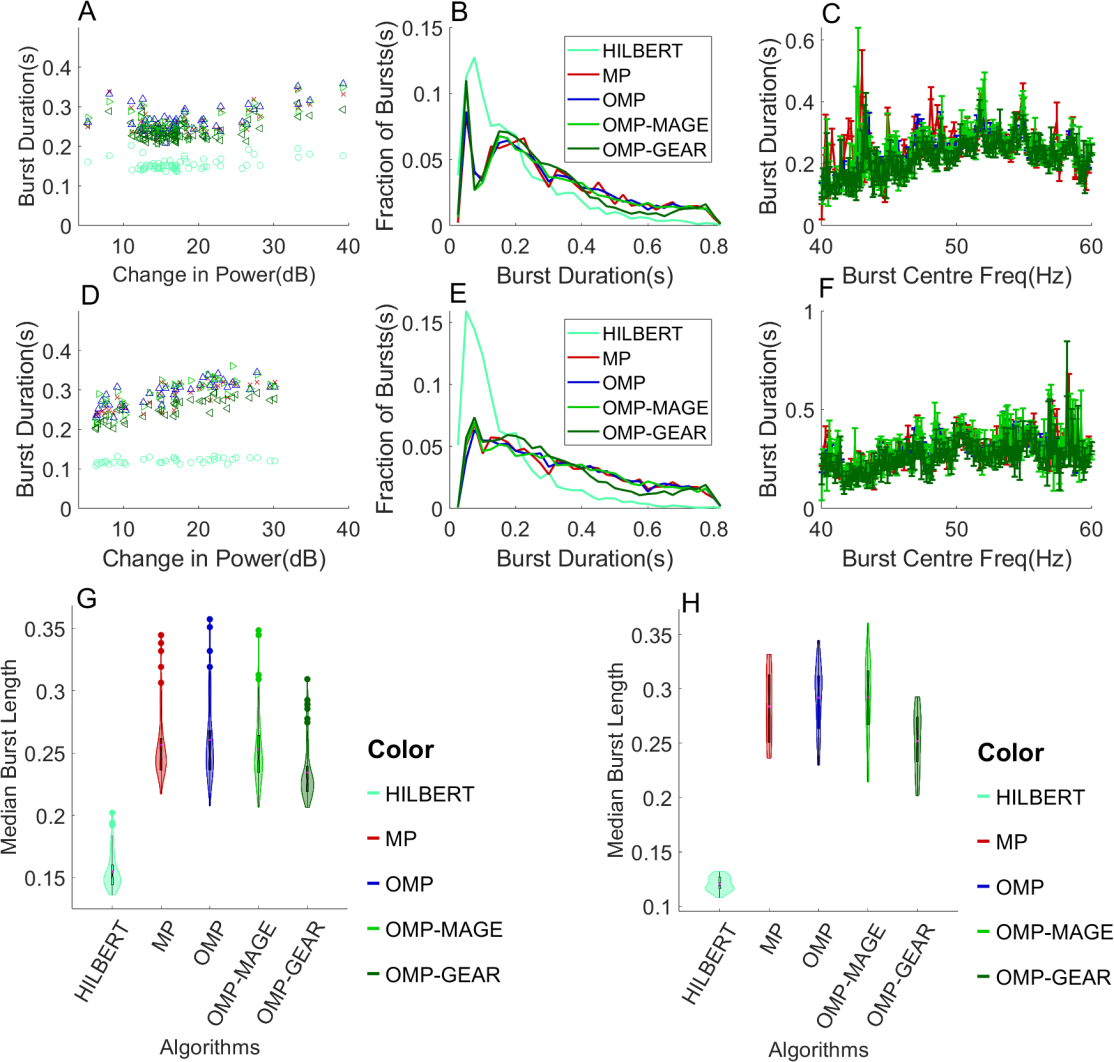
Gamma duration estimation from real LFP data. (A) Median gamma duration for each electrode as a function of the change in the gamma power relative to spontaneous activity for monkey 1, for the four methods. (B) Histogram of the gamma durations from all sites for monkey 1. (C) Median gamma duration returned at each unique frequency by MP, OMP, OMP-MAGE, and OMP-GEAR, for monkey 1. (D) Median gamma duration per site as a function of the change in power relative to the mean gamma power from spontaneous activity at that site for monkey 2. (E) Histogram of gamma duration from all sites for monkey 2. (F) Median gamma duration returned at each unique frequency by MP, OMP, OMP-MAGE, and OMP-GEAR, for monkey 2. (G) Violin plots embedded with box plots of median burst lengths for all algorithms, for monkey 1. (H) Violin plots embedded with box plots of median burst lengths for all algorithms, for monkey 2.

Finally, we estimated the burst durations from real LFP data recorded from two monkeys. Results are presented (see Fig. 5) in the same format as Subhash et al. (2018, figure 7). We set the threshold fraction 0.5 and used a dictionary size of 2.5 M for MP and 1.5 M for OMP, OMP-MAGE, and OMP-GEAR, as before. MP, OMP, and OMP-MAGE performed comparably, yielding median burst durations of 248.9 ± 2.97 ms, 248.9 ± 4.52 ms, and 246.5 ± 2.33 ms for monkey 1 and 287.2 ± 9.97 ms, 300.0 ± 4.85 ms, and 302.9 ± 7.85 ms for monkey 2, respectively. These durations were not significantly different from each other (p=0.173 , $\chi^{2}=3.51$ and p=0.39 , $\chi^{2}=1.86$ for monkeys 1 and 2, Kruskal–Wallis test when MP, OMP, and OMP-MAGE were considered). OMP-GEAR yielded slightly smaller durations (227.5 ± 1.27 ms for monkey 1 and 253.6 ± 9.8 ms for monkey 2) than OMP-MAGE ($p=3.1\times 10^{-6}$ , $\chi^{2}=21.75$ and $p=2.84\times 10^{-6}$ , $\chi^{2}=21.92$ for monkeys 1 and 2, pairwise Kruskal–Wallis test). The burst durations returned by the Hilbert-based method were much shorter (152 ± 1.45 ms and 120 ± 2.16 ms for monkey 1 and monkey 2, respectively).

## 4 Discussion

We implemented three methods for burst duration estimation: OMP, OMP-MAGE, and OMP-GEAR. The OMP method is a modified version of the original method (Pati et al., 1993), where we incorporate a phase correction step to enable OMP to identify a suitable (orthogonal) pair of atoms along with the correct phase in each iteration. OMP-MAGE combines the MAGE approach (Canolty & Womelsdorf, 2018) with OMP and incorporates the phase correction step mentioned above. Finally, we implemented a new method called OMP-GEAR. We found that when multiple bursts were presented, OMP-GEAR performed better than other methods in terms of correctly recovering the burst durations from synthetic data. All these methods gave longer median burst durations than traditional methods, validating the previous observation (using MP) that real data exhibit long burst durations (Subhash et al., 2018). The source codes and data are available at https://github.com/avianand8/GammaLengthProjectCodes. The list of implemented toolbox functions is provided in Appendix A.

### 4.1 Comparative performance of MP, OMP, OMP-MAGE, and OMP-GEAR

MP and OMP are greedy approaches for decomposing a signal as a linear combination of atoms from a dictionary. At each iteration, an atom is selected such that the energy in the residual signal decreases the most. The difference between MP and OMP lies in the least squares step in OMP, which makes the residue orthogonal to all the previously selected atoms. In turn, this results in the residual energy decreasing much faster in OMP than in MP. There are other approaches for atomic decomposition such as basis pursuit (S. Chen & Donoho, 1994), which pose atomic decomposition as a convex optimization problem.

We note that, in order to generate a dictionary of a given size, we first divide the parameter space into a fine dyadic grid, and generate atoms of the dictionary by sampling the grid points uniformly at random till the required number of unique atoms has been selected (see Section 2.3.1). The reason for using such a stochastic dictionary rather than a purely dyadic dictionary (which is generated by dividing the parameter space into a dyadic grid with the grid size such that the number of grid points equals the desired dictionary size, see Section 2.3.1) is because the latter introduces bias since the burst lengths are dyadic multiples of a base length. With OMP-MAGE and OMP-GEAR, we are not limited to the dictionary; the dictionary refinement step allows the algorithm to adjust the parameters of the atom to better explain the observation. Therefore, by carefully designing a fixed dictionary, OMP-MAGE and OMP-GEAR can yield better results even with small dictionary sizes.

From Figure 1, we see that all four methods perform similarly for burst durations exceeding about 0.1 s in the non-overlapping bursts case, and for burst durations exceeding about 0.2 s in the overlapping bursts case. This is because the number of bursts injected is small relative to the size of the dictionary.^*^ Due to this, all sparse recovery-based methods perform nearly the same. For shorter burst durations (around 0.1 s), the number of injected bursts becomes large, causing the algorithms to slightly overestimate the burst duration, especially in the overlapping bursts case, since bursts in proximity with similar phases could get merged as a single longer burst. At very low burst durations (around 50 ms), the number of bursts injected becomes too large (the mean number of bursts is 40) for sparse recovery techniques to work well, given the large size of the dictionary. A rule-of-thumb from the compressed sensing literature is that to recover an N-length, k-sparse vector using an m-dimensional linear observation obtained from noisy linear projections using an m×N measurement matrix, m=O(2klog N) is required. This condition fails when $N=1.5\times 10^{6}$, k=40 , since our measurements are m=1024 -dimensional vectors. Thus, all sparse recovery algorithms fail, as expected. Overall, this is an artifact of the way we have scaled the number of injected bursts as we reduce the injected burst length, but it helps to bring out the conditions under which the algorithms perform well and when they may fail.

Figure 2 shows that the orthogonal projections involved in the OMP-based algorithms allow them to pick sufficiently different atoms in subsequent iterations, resulting in a faster decay in the energy in the residue. Thus, OMP, OMP-MAGE, and OMP-GEAR converge much faster than the original MP algorithm. Also, for a given dictionary size, OMP-MAGE and OMP-GEAR are not limited to the atoms in the dictionary. If the initially selected atom is sufficiently close to a true atom, OMP-MAGE and OMP-GEAR are able to refine the dictionary itself and replace the atom with one that is much closer to the true atom. Hence, as the dictionary size is reduced (consequently, the algorithm speeds up), OMP-MAGE and OMP-GEAR continue to return an estimated burst length that is close to the injected burst length (300 ms in this case), while both MP and OMP significantly overestimate the burst length. Additionally, from Figure 5, we see that, on real data, MP performs comparably with the other three algorithms. This is because of the larger dictionary size (5 M) used with MP compared with the dictionary size (1.5 M) used with OMP and OMP-MAGE. OMP-GEAR yields slightly shorter burst durations, which could be because it is able to better resolve overlapping bursts, but these durations are still much longer than those obtained using Hilbert-based methods. Overall, atomic decomposition methods far outperform spectral methods such as the Hilbert transform-based method, showing that estimating the burst duration in the time domain itself is better than the frequency domain-based methods.

It must be noted that although atomic methods seemingly overestimate the burst durations occasionally, this is not necessarily incorrect, as there is ambiguity in the definition of a burst. If there are two nearby bursts that have a consistent phase and frequency relationship, they may be better represented by a single longer burst. Such a situation may arise physiologically if a sustained rhythm has an amplitude modulation, or if an underlying generator oscillates at a low frequency and generates bursts periodically (e.g., see van Ede et al., 2018, figure 1). Indeed, the power of atomic methods is that they can potentially capture rich dynamics containing multiple bursts that are phase aligned.

The regime where OMP-MAGE and OMP-GEAR outperform MP and OMP is when the number of injected bursts is moderately large (>10) and the dictionary size is relatively small (about 1 M). For much larger dictionary sizes (>5 M), the parameter reassignment used in OMP-MAGE and OMP-GEAR offers only a marginal advantage over OMP, as the dictionary already has sufficiently many atoms to represent the signal.

It is unclear why OMP-GEAR is able to better estimate the burst lengths than OMP-MAGE when multiple overlapping bursts are present. In both methods, we start with an initial estimate of an atom and subsequently refine it. OMP-MAGE uses a differentiation step (see Section 2.3.4) while OMP-GEAR (see Section 2.3.5) uses a different approach that skips the differentiation step. It is possible that skipping the differentiation step, which is prone to error or noise amplification, results in the improvement. In real data, we observe that the histograms in Figure 5B and E have a slightly lower fraction of bursts of durations around 500–600 ms and a slightly higher fraction around 200 ms compared with OMP-MAGE, suggesting that some bursts that were estimated as a single long burst by OMP-MAGE were instead resolved as two shorter bursts by OMP-GEAR. These observations lead us to believe that OMP-GEAR is a slightly better algorithm than OMP-MAGE, as it is able to resolve the bursts better.

### 4.2 Gamma bursts in the primary visual cortex

As discussed above, the BEAD algorithms perform very well in the presence of overlapping bursts. In our dataset, we observed such overlapping bursts frequently, with 78% of bursts showing at least a 50% overlap with another burst in the gamma range. The ability to identify overlapping bursts is especially important for gamma oscillations, since they are thought to be generated due to the interaction of excitatory and inhibitory neurons, with different inter-neuronal classes giving rise to gamma rhythms in slightly different frequency ranges; see (G. Chen et al., 2017; Veit et al., 2017). These oscillations are also hypothesized to interact (Veit et al., 2023; Wagatsuma et al., 2022), and the ability to capture these overlapping rhythms is important to study the nature of their interaction. For example, we found that the slow and fast gamma waves, thought to be generated by Somatostatin and Parvalbumin positive interneurons (G. Chen et al., 2017; Veit et al., 2017), respectively, form independent traveling waves even when they are overlapping in time (Gautham & Ray, 2024).

As discussed previously, MP-based decomposition leads to a better characterization of the fluctuations in the LFP when spikes are recorded. In particular, each component of the spike-related transient is well represented by a negative Gaussian, and removing it prior to the estimation of the phase of the rhythm to which the spikes may be phase locked allows better estimation of the phase at which spikes occur (Ray & Maunsell, 2015; Ray et al., 2008). This also helps in improving the computation of phase–amplitude coupling, where theta–gamma coupling observed in the primary visual cortex of monkeys was drastically reduced once the negative Gaussian was removed, suggesting that at least part of this coupling could be an artifact due to the spike-related transient (Prabhu & Ray, 2024). Characterization of such transients using overcomplete methods could potentially be useful to dissociate other structures in brain signals which also have high-frequency content, such as hippocampal sharp waves, which have distinct features (see Liu et al., 2022 for a detailed discussion).

While MP converges slowly compared with other methods (Fig. 2A), the difference in the rate of convergence is significant only after one or two orders of magnitude. However, the energy in brain signals falls with increasing frequency according to a power law, with the power in the high-gamma range and beyond being only a small fraction of the total signal energy. Due to this, to adequately capture the signal energy at high frequencies (up to a few hundred Hz), convergence down to a low residual energy is needed, and the convergence rate becomes important in these situations.

### 4.3 Burst estimation in neurophysiological data

In neurophysiological data, there are several issues that need to be addressed to properly identify burst events. For example, event-related potentials (ERPs) have sufficient power at low frequencies, which may be mistaken as oscillatory bursts, and hence need to be removed prior to burst estimation (Neymotin et al., 2022). More importantly, it is well known that the neural power spectra exhibit a “1/f” decay with frequency, and this aperiodic component needs to be separated from the oscillatory events that appear as bumps or spikes in the PSD. Brady and Bardouille developed an algorithm called periodic/aperiodic parametrization of transient oscillations (PAPTO) that disambiguates the aperiodic activity from the transient rhythmic bursts and showed that their approach captures more variance in the resting-state occurrence rate of beta events in a large MEG dataset compared with the more conventional transient event detection algorithms. Similarly, Szul et al. (2023) used the superlet transform (Moca et al., 2021) to generate single-trial time–frequency plots, to which they applied an iterative procedure to identify bursts based on an adaptive threshold for each iteration. Since our method works directly in the time domain, it is non-trivial to remove the aperiodic (1/f) component, which is estimated in the spectral domain. However, as shown in Figure 3B, BEAD methods are able to accurately identify the burst at even low threshold fractions, unlike classical methods (see Subhash et al., 2018, figure 4 for a comparison with other methods). The reason for this improved performance stems from the fact that, for classical methods, noise in the estimate of power (either due to noise in the spectral estimator or in the data) leads to an abrupt termination of the burst, which is more pronounced when the threshold fraction is low. For BEAD-based methods, however, the overall time domain signal structure is used to extract the burst, which naturally alleviates such issues.

Another issue is that the burst waveforms are not sinusoidal to begin with, but instead have stereotypical shapes. These shapes can be identified using techniques such as empirical mode decomposition (Quinn et al., 2021) or quantification of an individual oscillatory cycle by its amplitude, period, and waveform symmetry (Cole & Voytek, 2019). Even in the case of gamma oscillations, the waveform has a typical arch shape with sharper troughs and smoother peaks (Krishnakumaran & Ray, 2023). Thus, bursts need not have Gabor-like structures. One way to address this is to modify the dictionary such that the basis functions better represent the signal structure. For example, noting that beta bursts have a wavelet-like shape, Neymotin et al. (2022) used 7-cycle Morlet wavelets on nonoverlapping 10 s windows, with linearly spaced frequencies between 0.25 to 250 Hz with 0.25 Hz resolution. Subsequently, they normalized the power time series of each wavelet transform by the median power across the full recording duration and applied a local maximum filter to detect peaks that exceeded a threshold. Szul et al. (2023) also applied principal component analysis (PCA) on the identified burst waveforms to define a set of motifs that best explained the variance in these burst waveforms. Power et al. (2023) used convolutional dictionary learning (CDL) to detect transient bursts in a data-driven way and identified several task-related motifs that resembled bursts in beta, mu, and alpha ranges. These methods also capture signal structures related to artifacts such as eye movements or ECG, and, therefore, are useful for cleaning the data as well.

These methods contrast against our approach, where the dictionary is highly parametrized with only a few parameters describing the basis functions. Such parametrization has several benefits. Although non-parametrized application-specific methods can represent the signals with fewer atoms, their time–frequency analysis is not straightforward. However, the time–frequency representation of Gabor atoms is well known. More importantly, parameterized dictionaries allow us to develop methods such as OMP-MAGE and OMP-GEAR, which have low computational complexity and come with strong theoretical convergence guarantees. Further, Gabor atoms have the advantage that their Wigner–Ville distribution can be easily computed, and they have an optimal time–frequency trade-off. Parametrized dictionaries provide a simple way to extract features from a given data: Once an atom is found, its parameters immediately provide the features (position, frequency, and scale) of the atom, which would require post-processing in the case of non-parametrized methods. Due to this, the resulting identified atoms can potentially provide additional insights, for example, if the parameters have interesting relationships. Finally, a parametrized representation leads to efficient storage of the signals; for example, since Gabor atoms are completely determined by their parameters, we need to store only three parameters per atom.

Expanding parameterized dictionaries by including atoms that resemble non-sinusoidal structures (chirps, artifacts, atoms obtained in a data-driven fashion) is a worthwhile direction for future work.

### 4.4 Pros and cons of atomic decomposition-based methods

As seen previously, atomic decomposition-based methods can capture relationships between multiple bursts if they have some phase alignment. Also, depending on the signal characteristics, we can include other types of atoms in the dictionary such as chirp signals. Gamma oscillations are known to start with a high frequency on onset, which decays and stabilizes after some time. This transient behavior can potentially be better represented with chirps. Gamma oscillations may end more abruptly than the Gaussian window decay. One can include sinusoids multiplied by a rectangular window to capture these abrupt changes. The use of atomic decomposition and estimating the burst duration in the time domain provide the flexibility to include such atoms in the dictionary.

In terms of limitations, in our synthetic experiments, the injected bursts are Gabor waveforms and the dictionary that we used was also formed of Gabor atoms. The results could be different if injected bursts were generated using other waveforms but the dictionary was constructed using Gabor atoms. The greedy methods we have explored in this work have the limitation that if an incorrect atom is selected in a given step, the algorithms have no way of undoing the selection. Subsequent iterations that use a potentially incorrect residue may continue to pick incorrect atoms, cascading the error and resulting in a suboptimal signal representation. OMP-MAGE and OMP-GEAR alleviate this issue due to the dictionary refinement step, whereby these algorithms can correct for an incorrectly selected atom to some extent. However, it must be noted that even these algorithms require the initially selected atom to be sufficiently close to the true atom for the dictionary refinement to be effective. In turn, this means that the initial dictionary should sample the parameter space sufficiently finely to ensure that the dictionary contains an atom that is close to the true atoms. Finally, due to the large dictionary size requirement, these methods are computationally more expensive than traditional spectral decomposition-based methods.

## Data Availability

All codes and data required to perform the analysis in this manuscript are available on GitHub at https://github.com/avianand8/GammaLengthProjectCodes

## Footnotes

* The number of bursts is a Poisson distributed random variable whose mean equals the stimulus period (2 s in our case) divided by the burst duration.

## Appendix A: Toolbox Functions

**1. Omp_v1**

Implements the OMP algorithm. Required inputs: input signal (analogData), time values (timeVals), dictionary size (N1), and maximum number of iterations (itermax). Outputs: an array containing Gabor atom parameters (batoms), metadata about the signal and algorithm (header), signal reconstructed using the detected atoms (YFIT).

**2. Omp_gear**

Implements the OMP-GEAR algorithm. Required inputs: input signal (signal), dictionary size (dictSize), sampling rate (sRate), maximum number of iterations (itermax). Outputs: an array containing Gabor atom parameters (batoms), metadata about the signal and algorithm (header), signal reconstructed using the detected atoms (YFIT).

**3. reassign_gear**

Implements the GEAR reassignment function. Required inputs: initial Gabor atom parameters found by OMP (params_in), residual signal after the previous iteration of OMP (R), and sampling rate (sRate). Output: refined parameters (params_out).

**4. Omp_mage**

Implements the OMP-MAGE algorithm. Required inputs: input signal (signal), dictionary size (dictSize), sampling rate (sRate), maximum number of iterations (itermax). Outputs: an array containing Gabor atom parameters (batoms), metadata about the signal and algorithm (header), signal reconstructed using the detected atoms (YFIT).

**5. mageAtom_real and mageAtom_real**

Used for separating the real and imaginary parts of the Gabor atom defined by (10). Required inputs: time (t), frequency (v), scale (s), phase, sampling rate (sRate), and output signal length (L).

**6. makeDictionary_mage**

Generates a dictionary of Gabor atoms defined by (10). Required inputs: dictionary size (dictSize), sampling rate (sRate); input signal (signal) used to determine the length of the dictionary atoms.

**7. getStochasticDictionary_all**

Wrapper function to perform signal decomposition using any of the four algorithms: MP, OMP, OMP-MAGE, and OMP-GEAR. Inputs: a matrix whose rows contain the signals corresponding to each trial (data), a vector representing time values (timeVals), the maximum number of iterations to be used for the decomposition (maxIteration), and the size of the dictionary (dictionarySize). An adaptive dictionary parameter is only used by MP algorithm (adaptiveDictionaryParam). The algorithm to be used is specified using the parameter (algName). For example, for OMP, algName = “OMP”. Outputs: a matrix containing the extracted Gabor atoms parameters (gaborInfo) and a matrix containing metadata (header).

**8. getBurstLength_all**

The function extracts features such as burst length, burst frequencies, burst time from the given data. Required inputs: a matrix whose rows contain the time series corresponding to the signal recorded in each trial (analogData), a vector representing time values at which the signal was recorded (timeVals), threshold fraction (thresholdFactor), stimulus period (stimulusPeriodS), baseline period (baselinePeriodS), burst frequency range (burstFreqRangeHz), maximum number of iterations (maxIteration), dictionary size (dictionarySize), and the algorithm to be used (algName). Outputs: arrays containing burst duration estimated (lengthList), frequencies of the detected atoms (freqList), and burst time centers (timeList), a matrix containing parameters of detected atoms (gaborInfo), and a matrix containing metadata (header).

**9. testPerformanceSynthData_all**

Evaluates the performance of various burst detection algorithms on synthetic data. Inputs: A list containing threshold fractions used to compare algorithms (thresholdFractionList), the electrode number from which spontaneous LFP data are recorded (electrodeNum), mean number of bursts to be injected (numMeanBursts), dictionary size to be used (dictionarySize), algorithms to be compared (algNames). For example, algNames = [“MP” “OMP-MAGE” “HILBERT”].
